# TITAN: T-cell receptor specificity prediction with bimodal attention networks

**DOI:** 10.1093/bioinformatics/btab294

**Published:** 2021-07-12

**Authors:** Anna Weber, Jannis Born, María Rodriguez Martínez

**Affiliations:** IBM Research Europe, 8803 Rüschlikon, Switzerland; ETH Zurich, Department of Biosystems Science and Engineering (D-BSSE), 4058 Basel, Switzerland; IBM Research Europe, 8803 Rüschlikon, Switzerland; ETH Zurich, Department of Biosystems Science and Engineering (D-BSSE), 4058 Basel, Switzerland; IBM Research Europe, 8803 Rüschlikon, Switzerland

## Abstract

**Motivation:**

The activity of the adaptive immune system is governed by T-cells and their specific T-cell receptors (TCR), which selectively recognize foreign antigens. Recent advances in experimental techniques have enabled sequencing of TCRs and their antigenic targets (epitopes), allowing to research the missing link between TCR sequence and epitope binding specificity. Scarcity of data and a large sequence space make this task challenging, and to date only models limited to a small set of epitopes have achieved good performance. Here, we establish a *k*-nearest-neighbor (K-NN) classifier as a strong baseline and then propose Tcr epITope bimodal Attention Networks (TITAN), a bimodal neural network that explicitly encodes both TCR sequences and epitopes to enable the independent study of generalization capabilities to unseen TCRs and/or epitopes.

**Results:**

By encoding epitopes at the atomic level with SMILES sequences, we leverage transfer learning and data augmentation to enrich the input data space and boost performance. TITAN achieves high performance in the prediction of specificity of unseen TCRs (ROC-AUC 0.87 in 10-fold CV) and surpasses the results of the current state-of-the-art (ImRex) by a large margin. Notably, our Levenshtein-based K-NN classifier also exhibits competitive performance on unseen TCRs. While the generalization to unseen epitopes remains challenging, we report two major breakthroughs. First, by dissecting the attention heatmaps, we demonstrate that the sparsity of available epitope data favors an implicit treatment of epitopes as classes. This may be a general problem that limits unseen epitope performance for sufficiently complex models. Second, we show that TITAN nevertheless exhibits significantly improved performance on unseen epitopes and is capable of focusing attention on chemically meaningful molecular structures.

**Availability and implementation:**

The code as well as the dataset used in this study is publicly available at https://github.com/PaccMann/TITAN.

**Supplementary information:**

Supplementary data are available at *Bioinformatics* online.

## 1 Introduction

T-cells are an integral part of the adaptive immune system, whose survival, proliferation, activation and function are all governed by the interaction of their T-cell receptor (TCR) with immunogenic peptides (epitopes) presented on major histocompatibility complex molecules (MHC). A large repertoire of T-cell receptors with different specificity is needed to provide protection against a wide range of pathogens. This repertoire is generated using stochastic gene recombination and can theoretically produce diversities of 10^15^–10^20^ different receptors (Laydon et al., 2015) in an individual, each with unique binding capabilities. Due to this diversity, reliably predicting the binding specificity of a TCR from its sequence and understanding the mechanisms underlying TCR–pMHC interaction is highly challenging. At the same time, it has enormous potential to transform the field of immunology. A reliable prediction tool could unlock the wealth of information encoded in a patients’ TCR repertoire, which reflects their immune history and could inform about past and current infectious diseases, vaccine effectiveness or autoimmune reactions. Additionally, it could empower the application of therapeutic T-cells for cancer treatment, allowing the study of effectiveness and potential cross-reactivity risks *in silico*.

Recent advances in high-throughput sequencing techniques have led to the generation of an increasing amount of datasets linking TCR sequences to the epitopes they bind. However, the available data is still extremely sparse compared to the high dimensionality of the search space created by the TCR theoretical diversity. Moreover, current experimental settings typically link many TCRs to a single epitope, which leads to datasets that contain information about tens of thousands of TCRs, but only a few hundred different epitopes.

Nevertheless, several studies have attempted the prediction of TCR specificity from sequence using machine learning (for a review see Mösch et al., 2019). The most successful approaches so far are categorical epitope models, which exploit the relative abundance of TCR sequences to learn patterns of TCRs binding to the same epitope. Various machine learning concepts were applied to this task, including decision trees (De Neuter et al., 2018; Gielis et al., 2019), a range of different clustering approaches (Dash et al., 2017; Glanville et al., 2017; Jokinen et al., 2019) and Variational Autoencoders (Sidhom et al., 2021). Many of these can successfully predict which one of a small set of epitopes a given TCR will most likely bind to. However, these approaches are inherently incapable of predicting specificity to epitopes not contained in the training set (unseen epitopes), which fundamentally limits their applicability.

The next milestone toward this challenging goal are generic models, which explicitly encode both the TCRs and the epitopes. These have the potential to predict binding of any TCR–epitope pair, opening the door to the development of models that can generalize to both, unseen TCRs and epitopes. Current models show moderate performance on test data containing epitopes already encountered in training, but cannot extrapolate to unseen epitopes (Jurtz et al., 2018; Moris et al., 2020; Springer et al., 2019).

Tcr epITope bimodal Attention Networks (TITAN) exploits a bimodal neural network architecture to explicitly encode both TCR and epitope sequences. More specifically, TITAN uses convolutions to aggregate local information and fuses the modalities, using an interpretable attention mechanism from which binding probabilities are predicted.

Since interpretability is paramount in healthcare applications of machine learning, the use of context attention is central to our model, as it allows us to explain the choices of the algorithm and to analyze which amino acids or even atoms the model focuses on. These highlighted entities can be interpreted in the context of the underlying biochemical processes. We explore different encoding strategies for the epitopes such as SMILES (Weininger et al., 1989), a string-based, atom-level representation of molecules. SMILES are ubiquitously utilized in chemoinformatics for a wide range of applications, from predicting the chemical or pharmacological properties of molecules (Goh et al., 2017; Manica et al., 2019; Schwaller et al., 2018) to generative modeling (Gómez-Bombarelli et al., 2018). Using SMILES effectively results in a re-formulation of the TCR–epitope binding problem as the more general compound protein interaction (CPI) task, thus enabling the usage of large databases of protein-ligand binding affinity for pretraining, e.g. BindingDB including >1 M labeled samples (Gilson et al., 2016).

## 2 Materials and methods

### 2.1 Data

In order to assemble a larger and more diverse dataset, we combine data collected in the VDJ database (Bagaev et al., 2020) with a recently published COVID-19 specific dataset published by the ImmuneCODE project (Dines et al., 2020). Since paired chain data is still rare, we restrict ourselves to TCR*β* chain sequences.

We use all human TCR*β* sequences downloaded from the VDJ database (December 7, 2020), i.e. 40 438 TCR sequences assigned to 191 peptides. Since this dataset is highly imbalanced, we exclude epitopes with less than 15 associated TCR sequences and downsample to a limit of 400 TCRs per epitope. After these preprocessing steps, we are left with a dataset of 10 599 examples on 87 epitopes. We refer to this dataset as *VDJ*.

The COVID-19 dataset (published July 25, 2020) originally contained 154 320 examples associated with 269 different epitopes or groups of epitopes. To avoid ambiguity, we keep only samples associated with a single unique epitope and exclude unproductive sequences. Then we apply the same preprocessing steps as for the VDJ dataset, downsampling to 400 TCRs/epitope and excluding epitopes with less than 15 associated TCRs to arrive at a dataset of 12 996 examples.

We refer to the combined dataset with samples from VDJ and the COVID-19 dataset as *VDJ+COVID-19*.

Since these primary datasets contain only positive examples, we need to generate negative data. This can be achieved by shuffling the sequences, thereby associating TCRs with epitopes that they have not been shown to bind. Due to the low probability of a randomly drawn TCR binding a specific epitope, this manner of generating negative samples is established in the field (Fischer et al., 2020; Moris et al., 2020). It has also been shown to limit overestimation of performances in comparison to adding additional naive TCR sequences from other sources (Moris et al., 2020). Furthermore, by shuffling the pairing of TCRs and epitopes, we can match the number of negative examples to that of positive examples for each TCR, avoiding unbalanced datasets. With this procedure, we build a training dataset of 46 290 examples, 50% of which are positive, encompassing 192 different epitopes.

To ensure a fair comparison to the state of the art model ImRex, we also downloaded the publicly available dataset that ImRex was trained on. This dataset is based on the VDJ database and contains 13 404 samples for 118 different epitopes, with 50% negative samples. We use it to train all models for the final comparison (see Section 3.4). To evaluate the performances, we use an independent test set generated from the McPAS database (Tickotsky et al., 2017) (downloaded on November 3, 2020). We exclude non-human TCRs and remove all samples with TCRs contained in the ImRex training data. Then we split the McPas data into two test sets: one including all samples with epitopes contained in the ImRex training set (seen epitope test set) and one with epitopes not contained in the ImRex dataset (unseen epitope test set). For both test sets, 50% negative data is generated by shuffling. The final seen epitope test set contains 9740 samples, the unseen epitopes test set contains 1458 samples.

### 2.2 Models

#### 2.2.1 Problem formulation

We are interested in learning a mapping Φ between the space of receptors T and the space of epitopes E to the space of affinity scores A, i.e Φ:E×T→A. Φ is learned with a training dataset $D={\left\{e_{i},t_{i},a_{i}\right\}}_{i=1}^{N}$ where $e_{i}\in E,t_{i}\in T$ and $a_{i}\in \{0,1\}$ is a binary label indicating whether binding occurred.

#### 2.2.2 K-NN baseline

Our baseline model for the presented TCR-epitope binding prediction is constituted by a *k*-nearest-neighbor (K-NN) classifier. As a distance metric between samples, we utilize the sum of the length-normalized Levenshtein distance of the respective epitope and TCR protein primary sequences.

More formally, for the training data $D={\left\{e_{i},t_{i},a_{i}\right\}}_{i=1}^{N}$, we choose *e_i_* and *t_i_* to be epitope and TCR sequences, respectively (*t_i_* is the CDR3 region). Let $\left\{e_{j},t_{j}\right\}$ denote an unseen sample from the test dataset $D_{\mathrm{Test}}={\left\{e_{i},t_{i}\right\}}_{i=1}^{N_{\mathrm{Test}}}$. With the goal of predicting ${\hat{a}}_{j}$ to approximate the unknown *a_j_*, we first retrieve the subset of training data $D_{k}$ containing the *k* nearest neighbors using the distance measure

(1) $$D(e_{i},t_{i},e_{j},t_{j})=\frac{\mathrm{Lev}(e_{i},e_{j})}{\left|e_{j}\right|}+\frac{\mathrm{Lev}(t_{i},t_{j})}{\left|t_{j}\right|}$$

where |·| denotes sequence length and Lev(·,·) is the Levenshtein distance (Levenshtein, 1966), i.e. a string-based distance measure that measures the number of single-AA changes required to transform one sequence into the other. Then, the prediction ${\hat{a}}_{j}$ is trivially computed by ${\hat{a}}_{j}=\frac{\sum_{i}^{k}a_{i}}{k}$ with $a_{i}\in D_{k}$. We evaluate the model on all odd *k* (to avoid ties), s.t. 1≤k≤25, and choose the value for *k* leading to the best ROC-AUC score for comparisons. Note that this model is non-parametric.

### 2.3 Model architecture

**Fig. 1.**
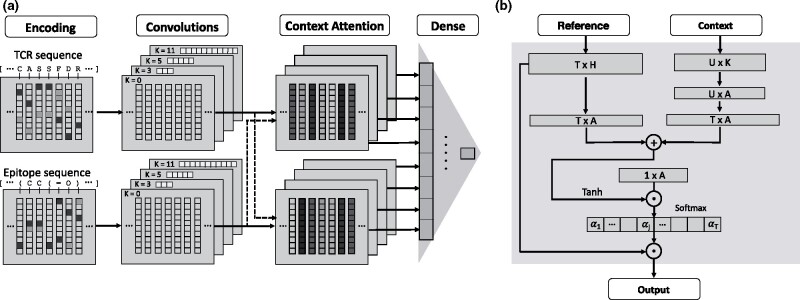
Overview of TITAN architecture. (**a**) Our model ingests a TCR and an epitope sequence, which get encoded using BLOSUM62 for amino acid sequences or learned embeddings for SMILES. Then, 1D convolutions of varying kernel sizes are performed on both input streams before context attention layers generate attention weights for each amino acid of the TCR sequence *given an epitope* and vice versa. Finally, a stack of dense layers outputs the binding probability. Conceptually, this architecture is identical to the one proposed in Born *et al.* (2021) (cf. Supplementary Fig. S3) but our visualization here is more fine-grained. (**b**) The linchpin of the model is the bimodal context attention mechanism. It ingests the convolved TCR and epitope encodings, treating one as *reference* and the binding partner as *context*. A series of transformations combines the modalities and yields an attention vector over the reference sequence (driven by the context) that can be overlayed with the molecule like a heatmap

Figure 1 shows an overview of the algorithmic steps of TITAN. To predict binding, we devise a bimodal architecture that separately ingests both the TCR and the peptide sequence.

The TCR sequences are encoded with the BLOSUM62 matrix (Henikoff and Henikoff, 1992), which is based on evolutionary similarity of amino acids and has been widely applied in TCR specificity prediction tools (Jurtz et al., 2018; Jokinen et al., 2019). Either the full sequence or only the CDR3 region was used as input. Since the antigenic peptides are small molecules, we explore two options to encode them: one that uses an amino acid-wise encoding such as BLOSUM62, and one that uses an atom-level encoding with SMILES. All sequences were padded to the same length of 500 tokens. Advantageously, SMILES representations of a molecule are not unique, thus facilitating data augmentation (Bjerrum, 2017).

The remaining architecture is inspired by Manica et al. (2019) and almost identical to the compound-protein-interaction (CPI) model presented in Born *et al.* (2021). In case of pretraining on CPI data (see below), the models are identical, otherwise, the SMILES-encoding ligand input stream is replaced with an AA-encoding epitope stream. Three parallel channels with convolutions of kernel sizes 3, 5 and 11 are employed on the input sequences to combine information from local neighborhoods of varying spatial extend. A fourth channel has a residual connection without convolutions (see Fig. 1a). For each of the four channels, we utilize two attention layers, where one modality is used as a context to compute attention scores over the other (see Fig. 1b). This allows the model to use information from the binding partner, i.e the context, to learn the importance of each token in the input sequence, i.e. the reference. The attention weights *α_i_* are computed as:

(2) $$\alpha_{i}=\frac{\;\text{exp}(u_{i})}{\sum_{j}^{T}\;\text{exp}(u_{j})}\;,\;\text{where}\;\vec{u}=\tanh\left(X_{1}W_{1}+W_{3}(X_{2}W_{2})\right)\vec{v}$$

We call $X_{1}\in R^{T\times H}$ the *reference* input, where *T* is the sequence length and *H* is the number of convolutional filters. Further, $X_{2}\in R^{U\times K}$ is the *context* input, where *U* and *K* are sequence length and number of convolutional filters in the other modality, respectively. $W_{1}\in R^{H\times A},\;W_{2}\in R^{K\times A},\;W_{3}\in R^{T\times U}$ and $\vec{v}\in R^{A}$ are learnable parameters. Intuitively, both inputs are projected into a common attention space $R^{A}$ with *A *=* *16 and then summed up, which enables the layer to take the context into account for determining feature relevance. $\vec{v}$ combines the information through a dot product, the output of which is fed to a softmax layer to obtain the attention weights *α_i_*, which are used to filter the inputs. Finally, both TCR and peptide information gets passed to two dense layers with 368 and 184 nodes, respectively, which output the binding probability.

### 2.4 Pretraining

By using SMILES encodings of the epitopes, predicting epitope receptor binding affinity can be seen as a CPI prediction task. We utilize BindingDB (Gilson et al., 2016), as of April 2020, to pretrain our model. To reduce the problem complexity and potential biases associated with the different experimental platforms to measure affinity, we binarize the binding data and define all entries in the database as binding, ignoring continuous affinity measurements. We generate an equal amount of negative examples by randomly assigning ligands to proteins (Born et al., 2021). In order to avoid high discrepancy between sequence lengths, ligands with a length > 250 SMILES tokens and proteins larger than 1028 amino acids are discarded. This results in 325 688 ligands and 3351 proteins and a total of 471 017 pairs from which 90% (423 915) are used for training and the rest for validation. To adapt the model size to the larger available dataset, we changed the layer sizes for pretraining by padding TCR sequences to 1028, setting the attention space *A *=* *256, using four convolutional channels with kernel sizes 3, 7, 9 and 13 for epitopes and 3, 7, 13, 19 for TCR and using three final dense layers with 2048, 1024 and 512 nodes.

### 2.5 Data splitting

**Fig. 2.**
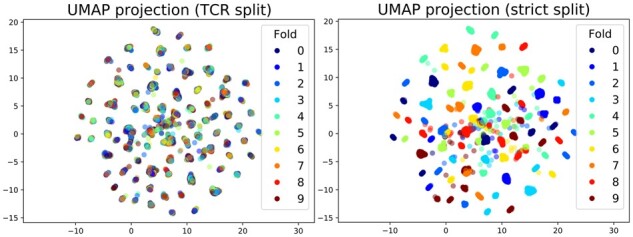
UMap Visualization of the VDJ dataset colored by the fold each sample belongs to in the TCR split (left) and strict split (right). The UMap projection leads to clear clusters of the samples. Coloring by fold in the TCR split reveals no connection between clusters and folds. However, coloring by fold in the strict split reveals that all the samples in a cluster belong to the same fold. This suggests that clusters correspond to distinct epitopes, highlighting their heterogeneity and the challenge of good generalization for the strict split.

We evaluate our models on a 10-fold cross-validation split. To determine the generalization capabilities of the models toward unseen TCRs and unseen epitopes separately, we use two different split methods. In the first, we ensure that each TCR is restricted to only one fold, which ensures that the validation datasets do not contain TCRs which were shown in training. The epitopes, however, are distributed randomly over the folds, so that most of the epitopes in the validation dataset were also shown during training. We refer to this split as the *TCR split*. Additionally, we generate a *strict split*, where we ensure that each TCR and each epitope is restricted to a single fold, ensuring that neither TCRs nor epitopes contained in the validation dataset were shown during training. To ensure the separation of TCRs and epitopes in their folds, we generate negative data by shuffling within each fold. A UMap (McInnes et al., 2018) visualization of all samples of the dataset is presented in Figure 2 and shows the ramifications of the splitting strategy. The feature space for the UMap dimensionality reduction was generated by embedding the amino acid sequences using a pretrained protein language model (Elnaggar et al., 2020).

### 2.6 Model training

All described architectures were implemented in PyTorch 1.4 and used the pytoda package for data handling and preprocessing. The models optimized binary cross entropy loss with Adam (Kingma and Ba, 2015) (*β*_1_ = 0.9, *β*_2_ = 0.999, *ϵ*  =  1e-8) and a learning rate of 0.0001. In the convolutional and dense layers, we employed dropout (*P *=* *0.5) and ReLU activation. All models were trained with a batch size of 512 on a cluster equipped with POWER8 processors and a single NVIDIA Tesla P100. The learning rate was tuned using the VDJ dataset and the remaining hyperparameters were chosen carefully based on previous experience.

## 3 Results

### 3.1 Performance on TCR split

**Fig. 3.**
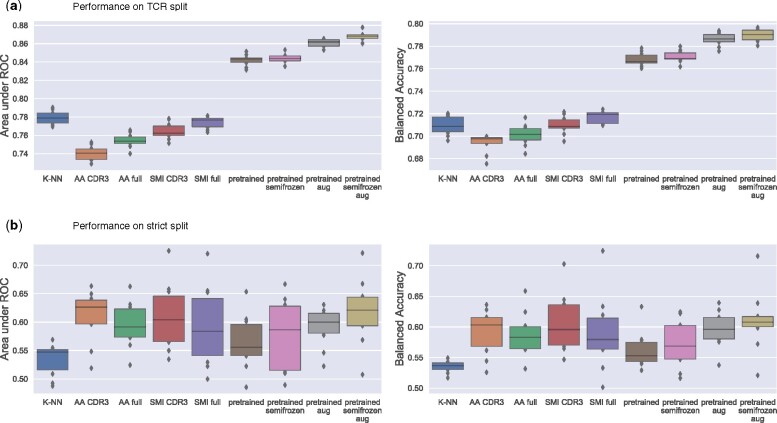
Performance comparison of different TITAN model settings trained or fine-tuned on the VDJ+Covid dataset. (**a**) ROC-AUC scores and balanced accuracy on a 10-fold crossvalidation TCR split (validation and training data share epitopes, but not TCRs). (**b**) ROC-AUC scores and balanced accuracy on a strict 10-fold crossvalidation split, where validation and training data share neither epitopes, nor TCRs. All boxplots: The center of each boxplot marks the sample median, and the box extends from lower to upper quartile. *K-NN* refers to the baseline model. Other abbreviations denote different settings under which TITAN was trained. *AA CDR3:* amino acid encoding of epitopes, only CDR3 sequence input for TCRs; *AA full:* amino acid encoding of epitopes, full sequence input for TCRs; *SMI CDR3:* SMILES encoding of epitopes, only CDR3 sequence input for TCRs; *SMI full:* SMILES encoding of epitopes, full sequence input for TCRs; *Pretrained:* SMILES encoding of epitopes, full sequence input for TCRs, model pretrained on BindingDB; *Pretrained semifrozen:* SMILES encoding of epitopes, full sequence input for TCRs, model pretrained on BindingDB, weights in epitope channel fixed during fine-tuning; *Pretrained aug:* SMILES encoding of epitopes, full sequence input for TCRs, model pretrained on BindingDB with data augmentation; *Pretrained semifrozen aug:* SMILES encoding of epitopes, full sequence input for TCRs, model pretrained on BindingDB with data augmentation, weights in epitope channel fixed during fine-tuning

In Figure 3a, we compare 10-fold crossvalidation performances of different TITAN settings on the TCR split scenario, which allows us to gauge the generalization capabilities of the algorithm toward unseen TCR sequences. We explored several options to input the sequence information of TCR and epitope in our model. Following the well-established concept of word embeddings (Mikolov et al., 2013), we can encode the amino acids using a fixed-length vector representation (in our case 32-dimensional), that is initialized randomly and then learned through training. Alternatively, we can represent each amino acid as a vector containing biophysical properties (e.g. molecular weight, residue weight, pKa, pKb, pKx, pI and hydrophobicity at pH2). Finally, we explored using evolutionary substitution matrices like BLOSUM62. Each row in the BLOSUM matrix represents the probability for an amino acid to be substituted by any of the other amino acids and can be used as a 26-dimensional vector representation of that amino acid.

An initial comparison of these embedding options showed no clear preference for either of them (See Table 1). Since a learned embedding is less reproducible and biophysical feature choices can be debated, we decided to use the BLOSUM62 matrix to embed amino acid sequences in all model settings.

**Table 1.** Comparison of amino acid embeddings

Embedding	ROC-AUC
Biophysical features	0.76 ± 0.01
Learned embedding	0.75 ± 0.01
BLOSUM62 matrix	0.75 ± 0.01

*Note*: Mean and standard deviation of the *AA CDR3* model configuration on 10-fold TCR split of the VDJ dataset. All tested amino acid embeddings show a similar performance.

For TCRs, we have the options to either focus solely on the hypervariable CDR3 loop, which is known to be the main peptide binding region, or to consider the full variable region of the TCR*β* sequence, which includes the V, D and J segments and encompasses all three hypervariable loops CDR1, CDR2 and CDR3. Figure 3a shows that the use of the full sequence information boosts the model performance, indicating that valuable information is contained in regions outside of the CDR3 loop.

Since epitopes are relatively short (5 to 15 amino acids), we can represent them in a fine-grained, atom-wise manner using SMILES strings. We can see in Figure 3a that the SMILES representation of epitopes further improves performance compared to an amino acid encoding.

The combination of SMILES for epitopes and full sequence encoding of the TCR leads to a mean ROC-AUC of 0.77 ± 0.006 and a mean balanced accuracy of 0.72 ± 0.005. However, we see that even this improved model does not outperform the simple K-NN baseline model that we included as a comparison. With *k *=* *13, the K-NN baseline achieves the best results with a ROC-AUC of 0.78 ± 0.007 and a balanced accuracy of 0.71 ± 0.008 (see Supplementary Fig. S1). This highlights the importance of including an appropriate baseline model, which is so far rarely observed in the field, as simple models may outperform complex ones in a sparse data setting. We emphasize that this is not an argument *against* our neural networks, but *for* the K-NN baseline model, which also outperforms the state of the art model, ImRex, a recent approach that uses 2D CNNs (Moris et al., 2020) (see Section 3.4 for a more detailed comparison of TITAN and ImRex). We also note that an approach similar to our K-NN baseline, TCRMatch (Chronister et al., 2020), was recently presented in a preprint. TCRMatch predicts TCR specificity using only sequence similarities to previously characterized receptors.

All model comparisons on the TCR split are also summarized in Table 2. For better comparability to previously published models, we also include scores obtained on the dataset excluding the samples gathered from the COVID-19 dataset.

**Table 2.** Tenfold cross validation performance on TCR split

	ROC-AUC	
Model	VDJ	VDJ+COVID-19
K-NN (Baseline)	0.79 ± 0.01	0.78 ± 0.007
AA CDR3	0.75 ± 0.02	0.74 ± 0.007
AA full	0.76 ± 0.007	0.75 ± 0.007
SMI CDR3	0.73 ± 0.007	0.76 ± 0.008
SMI full	0.75 ± 0.006	0.77 ± 0.006
Pretrained	0.81 ± 0.01	0.84 ± 0.005
Pretrained aug.	0.80 ± 0.01	0.86 ± 0.004
Pretrained semifrozen	0.80 ± 0.01	0.84 ± 0.005
Pretrained semifrozen aug.	0.82±0.01	0.87±0.005

*Note*: Mean and standard deviation of each model configuration on the VDJ dataset and the VDJ + COVID-19 dataset. Best performance marked in bold.

Furthermore, the results in Table 2 also indicate that the available interaction data may be too sparse to enable the models to learn the complex interaction patterns governing TCR–epitope binding. However, using the SMILES encoding for epitopes and the full sequence encoding for TCRs, we have effectively re-formulated the task as a compound protein interaction, which allows us to use BindingDB (Gilson et al., 2016) to pretrain the model, before we fine-tune it on the TCR–epitope interaction data. The pretrained model performed well on the held-out data from BindingDB (ROC-AUC 0.895).

Regarding the pretraining, we tested two different settings, one where all weights could be adapted during fine-tuning, and a *semifrozen* setting, where we only allowed weight changes in the TCR channel and the final dense layers of the epitope. This was done to prevent the model from ‘unlearning’ to recognize relevant SMILES features due to the extremely low number of different epitopes in the fine-tuning dataset. Figure 3 shows that pretraining severely improves model performance in both settings. We further improved model performance by exploiting the non-uniqueness of SMILES strings to perform data augmentation. Augmentation is achieved by randomly generating a valid SMILES representation of the epitope on the fly at each training step (Born et al., 2021b). The best overall model performance was achieved by the semifrozen pretrained model with augmentation, with a mean ROC-AUC of 0.87 ± 0.005 and a mean balanced accuracy of 0.79 ± 0.005, clearly outperforming the K-NN baseline by a large margin.

The high performance on validation data that does not contain TCR sequences used in training shows that the model successfully generalizes to unseen TCRs. Comparing the performance across groups of TCRs with different similarities to the training data, we find that while the model performs better for TCRs that are highly similar to their closest partners in the training set, the performance on the TCRs with highest distance from the training data is still high (ROC AUC 0.84 ± 0.016, balanced accuracy 0.71 ± 0.008, see Supplementary Fig. S3). Moreover, it is interesting to observe that models pretrained on the BindingDB (Gilson et al., 2016) performed better on the larger and more heterogeneous dataset VDJ + COVID-19, which suggests that pretraining might not only increase performance, but also enable the model to better generalize to different datasets.

### 3.2 Analysis of attention layers

**Fig. 4.**
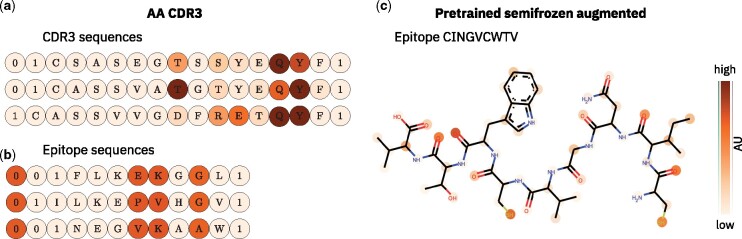
Analysis of the context attention layers. The attention weights *α_i_* on each token are extracted from the context attention layer and represented as a colormap. In (**a**) and (**b**), examples of the *AA CDR3* setting are shown, where epitopes are input as amino acid sequences and only CDR3 sequences are input in the TCR channel. 0 denotes the padding and 1 the <START> and <STOP> token. (a) The attention scores over three exemplary CDR3 sequences. The model adapts the attention scores based on the different inputs. (b) The attention on three exemplary epitopes. The model fails to adapt the attention scores to different inputs. (**c**) Attention scores over an exemplary epitope that was not included in the training dataset. The model was pretrained on BindingDB and fine-tuned on the training data with a frozen epitope input channel with SMILES augmentations to enrich the data. The lower bound of the color scale was set to one standard deviation above the mean attention on the padding tokens.

We can investigate the decision processes of TITAN using the information contained in the attention layers. Figure 4a shows the attention scores of TITAN in the *AA CDR3* setting—a setting that clearly outperforms previous work (see Section 3.4)—over a number of exemplary CDR3 sequences. We see that while there is some preference to focus on certain positions, the model adapts the attention to the different sequences. The mean inter-TCR variance per token is at $4.3\times 10^{-5}$. Moreover, we find that the attention also adapts to the context (i.e. the epitope), for which we want to predict the interaction. The mean variance per token within the same TCR interacting with different epitopes is $1.3\times 10^{-5}$. This demonstrates that the model is capable of adapting the attention layer to both the input and the context.

Figure 4b shows the attention of the same model (*AA CDR3* setting) on several exemplary epitopes. We can clearly see that the model chooses to focus heavily on the same positions on each epitope. The preferred positions are independent of the sequence of both the input epitope and the context TCR. Comparing the attention scores across epitopes, we find that both the inter-epitope and the intra-epitope variance of the attention are extremely small, at $4.9\times 10^{-10}$ and $2.5\times 10^{-9},$ respectively. This behavior indicates that TITAN fails to learn meaningful patterns in the epitope sequences from the limited diversity of epitopes in the dataset. We conjectured that the model finds a way to internally generate classes of epitopes represented by meaningless—but unique—vectors and predict specificity of TCRs toward these. This hypothesis is supported by the observation that compressing each epitope sequence to the chain of amino acids with attention scores $\alpha_{i}>0.1$ yields only a very moderate reduction in the number of unique sequences. Crucially, these shortened epitope sequences are still unique for 185 out of 192 epitopes in the dataset (96%). By focusing on these fixed, invariant positions the model circumnavigates learning generic representations of epitopes and instead internally classifies epitopes, at the cost of losing the power to differentiate 19 epitopes.

Pretraining the models on the compound interaction task greatly increased the diversity of sequences that were seen by the epitope channel of the model. While most of the ligands in BindingDB are not peptides, and may therefore exhibit structures and chemical properties that differ strongly from the epitopes, the model may nevertheless infer general rules of chemical interaction from them. This enables the model to learn more meaningful attention weights, which may be the reason for the performance boost we observe for pretrained models. In Figure 4b, we show a visual representation of the attention scores over one exemplary epitope as a case study of TITAN in the *pretrained semifrozen augmented* setting. The chosen epitope CINGVCWTV was not shown during training. The attention scores therefore reflect the transferable knowledge the model has gained during pretraining. We see that the attention patterns align well with our expectations. The attention is high on many of the oxygens, as well as on the two thiol groups of the cysteines. Nevertheless, we see that while the attention layers may extract some chemically relevant structures, they fail to capture others. Specifically, large and hydrophobic amino acid sidechains tend to get low attention scores, although they may be of high importance for molecule interactions.

### 3.3 Performance on strict split

Figure 3b compares the performances of the different TITAN settings on the strict epitope split, which measures the generalization capabilities to unseen epitopes. As expected, model performance drops severely across all settings. Moreover, we find that all TITAN settings perform similarly, with mean ROC-AUC scores around 0.6, while the K-NN baseline model shows a mean ROC-AUC of only 0.54 ± 0.03 for a choice of *k *=* *25 (see Supplementary Fig. S2 for comparison). Compared to the unseen TCR performance, we also see an increase in the standard deviation of the scores, with ROC-AUC scores of over 0.7 for some folds, and 0.5 for others. All mean ROC-AUC values are summarized in Table 3.

**Table 3.** Tenfold cross validation performance on strict split

Model	ROC-AUC
K-NN (Baseline)	0.54 ± 0.03
AA CDR3	0.60 ± 0.04
AA full	0.59 ± 0.04
SMI CDR3	0.60 ± 0.06
SMI full	0.59 ± 0.06
Pretrained	0.56 ± 0.04
Pretrained + Aug.	0.59 ± 0.03
Pretrained semifrozen	0.58 ± 0.06
Pretrained semifrozen + Aug.	0.62±0.06

*Note*: Mean and standard deviation of each model configuration on the VDJ + COVID-19 dataset. Best performance marked in bold.

The best score overall is still achieved by the *pretrained semifrozen augmented* model, with a mean ROC-AUC of 0.62 ± 0.06 and a mean balanced accuracy of 0.61 ± 0.05. However, its superiority over other TITAN setting is not as large as in the TCR split scheme. We can also see that pretraining does not strongly improve model performance on unseen epitopes.

**Fig. 5.**
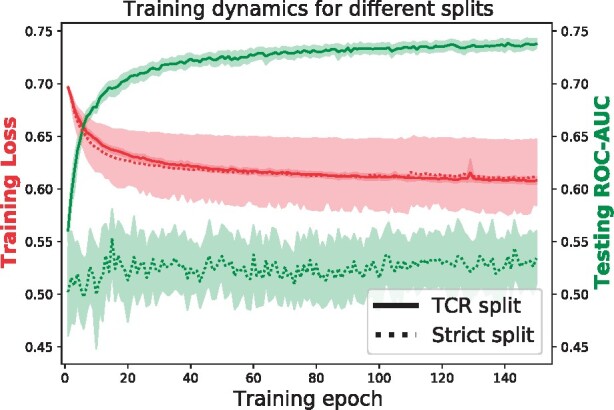
Training dynamics for both splitting strategies. A key distinction between the training on the TCR and strict splits is that the validation performance steadily converges only in the TCR split case. In the strict split case, no significant improvement is achieved even after training for more than 140 epochs. This indicates that in the strict split scenario, training and validation data may be too distinct to enable a proper generalization. The AA CDR3 setting was used for this plot.

Another surprising result is the comparably good performance of the TITAN *AA CDR3* setting on unseen epitopes. The analysis of the attention layer in Figure 4a shows that in this setting, TITAN uses an attention mask to reduce the task to a TCR classification problem. This should prevent the model to generalize to new epitopes. A close look at Figure 5 reveals that the best performance of the *AA CDR3* model on the strict split is achieved during the first 10 to 20 epochs of training. During this time, the attention is still uniformly distributed over all input tokens, because many training epochs are required for the model to build the static attention mask described in Section 3.2.

Figure 5 also shows that while the ROC-AUC increases continuously over the training epochs in the TCR split cross-validation, it stagnates during training on the strict split. We hypothesize that the underlying factor causing this phenomenon is the violation of the i.i.d. assumption across training and validation data. This behavior was common during training on the strict split and again highlights the challenge for models to generalize to unseen epitopes from the sparsity of the current datasets.

While TITAN’s generalization capabilities to unseen epitopes are still limited, the performance is moderately good. This suggests that, despite their sparsity, the currently available datasets do contain enough information to enable generalization to unseen epitopes to a certain degree.

### 3.4 Comparison to ImRex model on independent test set

Moris *et al.* recently published ImRex (Moris et al., 2020), an image-based generic TCR specificty prediction model, which explicitly encodes epitopes and can make predictions for unseen epitopes. As a direct comparison to ImRex, we trained TITAN with different settings on the ImRex training data and tested performance on an independent test set generated from the McPAS database (see Section 2.1). To judge both generalization capabilities to unseen TCRs and unseen epitopes, we used two different subsets of the McPAS data, one where all samples containing TCRs from the ImRex training dataset were excluded (*seen epitopes* test set) and one where all samples including epitopes contained in the ImRex training dataset were removed (*unseen epitopes* test set). ROC-AUC scores are compared in Table 4. We emphasize that this is not a cross-validation setting, i.e. we train on the full training set and record performance on an independent test set.

**Table 4.** Comparison of TITAN with prior work

	ROC-AUC	
Model	Seen epitopes	Unseen epitopes
ImRex	0.61	0.50
K-NN (Baseline)	0.79	0.37
AA CDR3	0.83	0.69
AA full	0.81	0.64
SMI CDR3	0.85	0.72
SMI full	0.86	0.64
Pretrained	0.79	**0.78**
Pretrained + Aug.	0.83	0.65
Pretrained semifrozen	0.77	0.69
Pretrained semifrozen + Aug.	**0.87**	0.60

*Note*: All but the ImRex model (shaded in gray) are contributions of this work. Models were trained on identical data and tested on an independent test set to ensure a fair comparison. Best performance marked in bold.

We find that all of our model settings outperform ImRex on both independent test sets by a large margin. Moreover, our K-NN baseline model clearly outperforms ImRex on seen epitopes.

Moreover, we see that the base models (*AA CDR3, AA full, SMI CDR3, SMI full*) all perform better on the independent *seen epitopes* test data than during 10-fold crossvalidation, while for the pretrained model settings, performance is comparable to the crossvalidation. For the *unseen epitopes* test set, we observe a similar trend as above in the strict split cross-validation. All models show performances clearly better than chance, with the pretrained semifrozen model with augmentation even achieving a ROC-AUC of 0.78. However, one needs to keep in mind, that the sample size for the *unseen epitopes* test is at only 1500, making especially high (or low) scores likely to be statistical outliers.

## 4 Discussion

In this work, we present TITAN, a generic, bimodal, sequence-based neural network for predicting TCR–epitope binding probability that significantly outperforms the state-of-the-art. We compare several settings of TITAN that differ in their inputs for TCRs and epitopes. While we restrict ourselves to the TCR*β* chain, we find that inputting the complete variable TCR sequence boosts performance compared to scenarios where only the CDR3 region is used.

Notably, we are, to the best of our knowledge, the first to formulate TCR-epitope binding prediction as a subclass of the commonly investigated task of predicting compound-protein-interaction. The concomitant change of representing epitopes as SMILES (an atomic, more granular representation) instead of amino acid sequences improves the predictive power of TITAN. This reformulation not only enables data augmentation—by exploiting the non-uniqueness of SMILES (Bjerrum, 2017)—to enrich the training data but also positions us to leverage large-scale datasets such as BindingDB for pretraining. Pretrained TITAN models achieve considerably higher scores than all TITAN base models. Freezing the weights of the epitope input channel coupled with SMILES augmentation gives us the best performance with a mean ROC-AUC of 0.87 ± 0.005 on the TCR split.

To assess model performance in a more rigorous fashion, we designed a K-NN classifier based on the Levenshtein distance as a baseline model. Surprisingly, this simple model achieves a mean ROC-AUC score of 0.79 ± 0.007 on the TCR split crossvalidation. This model surpassed the performance of complex neural networks from previous publications (Moris et al., 2020) and is only outperformed by our pretrained TITAN models, which highlights the importance of including relevant baseline models.

As a final assessment of model performance, we compare TITAN to the current state of the art for general TCR specificity prediction, ImRex. To ensure fair comparison, we train our models on the ImRex training data and evaluate the performance on an independent test set derived from a different database. We demonstrate that both pretrained and base TITAN models, as well as the K-NN baseline, outperform ImRex by a large margin. The best result is again achieved by the pretrained semifrozen augmented TITAN model, with a ROC-AUC of 0.87 on epitopes included in the training data.

Finally, the main challenge for generic TCR–epitope interaction prediction remains the generalization to unseen epitopes. Here, we report two major breakthroughs. First, we demonstrate that TITAN exhibits moderate performance on unseen epitopes, where the best TITAN model achieves a ROC-AUC of 0.62 ± 0.05 on a strict 10-fold crossvalidation split, and ROC-AUC of 0.78 on an independent test set of unseen epitopes. Second, by dissecting the attention heatmaps we were able to identify a possible explanation for the observed poor unseen epitope generalization capabilities. We demonstrate that TITAN reduces the general TCR–epitope prediction task to the simpler task of TCR classification, by internally treating the epitopes as classes instead of focusing on their properties. This shortcut could present a general problem for sufficiently complex models, as long as the extreme sparsity of sampling of the epitope sequence space is not remedied. Until then, our future endeavors might include using an enriched training dataset consisting of TCR-epitope pairs as well as compound-protein interaction pairs from BindingDB, or further improving the amino acid and SMILES embeddings by training on diverse databases (UniProt Consortium, 2020; Gaulton et al., 2017). In general, our results indicate that approaches with a focus on leveraging transfer learning techniques to enrich the input data space may be promising to tackle the daunting task of unseen epitope-TCR specificity prediction.

## Supplementary Material

btab294_Supplementary_Data

## References

[btab294-B1] Bagaev D.V. et al (2020) VDJdb in 2019: database extension, new analysis infrastructure and a T-cell receptor motif compendium. Nucleic Acids Res., 48, D1057–D1062. 31588507 10.1093/nar/gkz874 PMC6943061

[btab294-B2] Bjerrum E.J. (2017) SMILES enumeration as data augmentation for neural network modeling of molecules. http://arxiv.org/abs/1703.07076.

[btab294-B3] Born J. et al (2021) Data-driven molecular design for discovery and synthesis of novel ligands – a case study on sars-cov-2. Mach. Learn. Sci. Technol., 2, 025024.

[btab294-B4] Born J. et al (2021) PaccMann^RL^: de novo generation of hit-like anticancer molecules from transcriptomic data via reinforcement learning. iScience, 24, 102269. 33851095 10.1016/j.isci.2021.102269 PMC8022157

[btab294-B5] Chronister W.D. et al (2021) TCRmatch: Predicting T-cell Receptor Specificity based on Sequence Similarity to Previously Characterized Receptors. Front. immunol., 12, 673. 10.3389/fimmu.2021.640725 PMC7991084 33777034

[btab294-B6] Consortium T.U. (2020) UniProt: the universal protein knowledgebase in 2021. Nucleic Acids Res., 49, D480–D489. 10.1093/nar/gkaa1100 PMC7778908 33237286

[btab294-B7] Dash P. et al (2017) Quantifiable predictive features define epitope-specific T cell receptor repertoires. Nature, 547, 89–93. 28636592 10.1038/nature22383 PMC5616171

[btab294-B8] Neuter N.D. et al (2018) On the feasibility of mining CD8+ T cell receptor patterns underlying immunogenic peptide recognition. Immunogenetics, 70, 159–168. 28779185 10.1007/s00251-017-1023-5

[btab294-B9] Dines J.N. et al (2020) The immunerace study: a prospective multicohort study of immune response action to covid-19 events with the immunecode^TM^ open access database. medRxiv.

[btab294-B10] Elnaggar A. et al (2020) Prottrans: towards cracking the language of life’s code through self-supervised deep learning and high performance computing. CoRR, abs/2007.06225, https://arxiv.org/abs/2007.06225

[btab294-B11] Fischer D.S. et al (2020) Predicting antigen specificity of single t cells based on TCR cdr3 regions. Mol. Syst. Biol., 16, e9416. 32779888 10.15252/msb.20199416 PMC7418512

[btab294-B12] Gaulton A. et al (2017) The ChEMBL database in. Nucleic Acids Res., 45, D945–D954. 27899562 10.1093/nar/gkw1074 PMC5210557

[btab294-B13] Gielis S. et al (2019) Detection of enriched T cell epitope specificity in full T cell receptor sequence repertoires. Front Immunol., 10, 2820. 31849987 10.3389/fimmu.2019.02820 PMC6896208

[btab294-B14] Gilson M.K. et al (2016) Bindingdb in 2015: a public database for medicinal chemistry, computational chemistry and systems pharmacology. Nucleic Acids Res., 44, D1045–D1053. 26481362 10.1093/nar/gkv1072 PMC4702793

[btab294-B15] Glanville J. et al (2017) Identifying specificity groups in the T cell receptor repertoire. Nature, 547, 94–98. 28636589 10.1038/nature22976 PMC5794212

[btab294-B16] Goh G.B. et al (2017) SMILES2Vec: an interpretable general-purpose deep neural network for predicting chemical properties. CoRR, abs/1712.02034, http://arxiv.org/abs/1712.02034.

[btab294-B17] Gómez-Bombarelli R. et al (2018) automatic chemical design using a data-driven continuous representation of molecules. ACS Central Sci., 4, 268–276. 10.1021/acscentsci.7b00572 PMC5833007 29532027

[btab294-B18] Henikoff S. , Henikoff J.G. (1992) Amino acid substitution matrices from protein blocks. Proc. Natl. Acad. Sci. USA, 89, 10915–10919. 1438297 10.1073/pnas.89.22.10915 PMC50453

[btab294-B19] Jokinen M. et al (2021) TCRGP: determining epitope specificity of T cell receptors. PLoS Comput Biol, 17(3), e1008814. 10.1371/journal.pcbi.1008814 PMC8023491 33764977

[btab294-B20] Jurtz V.I. et al (2018) NetTCR: sequence-based prediction of TCR binding to peptide-MHC complexes using convolutional neural networks. bioRxiv. 433706, doi: 10.1101/433706.

[btab294-B21] Kingma D.P. , Ba J.L. (2015) Adam: a method for stochastic optimization. In: *3rd International Conference on Learning Representations*, San Diego, CA, USA, ICLR 2015 – Conference Track Proceedings. https://arxiv.org/abs/1412.6980v9.

[btab294-B22] Laydon D.J. et al (2015) Estimating T-cell repertoire diversity: limitations of classical estimators and a new approach. Philos. Trans. R. Soc. B Biol. Sci., 370, 20140291. 10.1098/rstb.2014.0291 PMC4528489 26150657

[btab294-B23] Levenshtein V. (1966) Binary codes capable of correcting deletions, insertions, and reversals. Soviet Physics Doklady, 10, 707–710.

[btab294-B24] Manica M. et al (2019) Toward explainable anticancer compound sensitivity prediction via multimodal attention-based convolutional encoders. Mol. Pharm., 16, 4797–4806. 31618586 10.1021/acs.molpharmaceut.9b00520

[btab294-B25] McInnes L. et al (2018) Umap: uniform manifold approximation and projection for dimension reduction. CoRR, abs/1802.03426, https://arxiv.org/abs/1802.03426.

[btab294-B26] Mikolov T. et al (2013) Efficient estimation of word representations in vector space. In: 1st International Conference on Learning Representations, Scottsdale, Arizona, USA, ICLR 2013 - Workshop Track Proceedings. https://arxiv.org/abs/1301.3781.

[btab294-B27] Moris P. et al (2020) Current challenges for unseen-epitope TCR interaction prediction and a new perspective derived from image classification. Brief. Bioinf., bbaa318, 1477–4054 10.1093/bib/bbaa318 PMC8294552 33346826

[btab294-B28] Mösch S. et al (2019) Machine learning for cancer immunotherapies based on epitope recognition by T cell receptors. *Front. genet*., 10, 1141. 10.3389/fgene.2019.01141 PMC6878726 31798635

[btab294-B29] Schwaller P. et al (2018) Found in translation: predicting outcomes of complex organic chemistry reactions using neural sequence-to-sequence models. Chem. Sci., 9, 6091–6098. 30090297 10.1039/c8sc02339e PMC6053976

[btab294-B30] Sidhom J.-W. et al (2021) DeepTCR is a deep learning framework for revealing sequence concepts within T-cell repertoires. Nat. Commun., 12, 1–12. 33707415 10.1038/s41467-021-21879-w PMC7952906

[btab294-B31] Springer I. et al (2020) Prediction of specific TCR-peptide binding from large dictionaries of TCR-peptide pairs. Front. immunol., 11, 1803. 32983088 10.3389/fimmu.2020.01803 PMC7477042

[btab294-B32] Tickotsky N. et al (2017) Mcpas-TCR: a manually curated catalogue of pathology-associated t cell receptor sequences. Bioinformatics, 33, 2924–2929. 28481982 10.1093/bioinformatics/btx286

[btab294-B33] Weininger D. et al (1989) SMILES. 2. algorithm for generation of unique SMILES notation. J. Chem. Inf. Comput. Sci., 29, 97–101.

