# Supplementary figures and images for: TITAN: T-cell receptor specificity prediction with bimodal attention networks
Source: Bioinformatics. 2021 Jul 12;37(Suppl 1):i237–44. doi: 10.1093/bioinformatics/btab294 (PMC8275323; doi:10.1093/bioinformatics/btab294)

# KNN - VDJ Validation performance

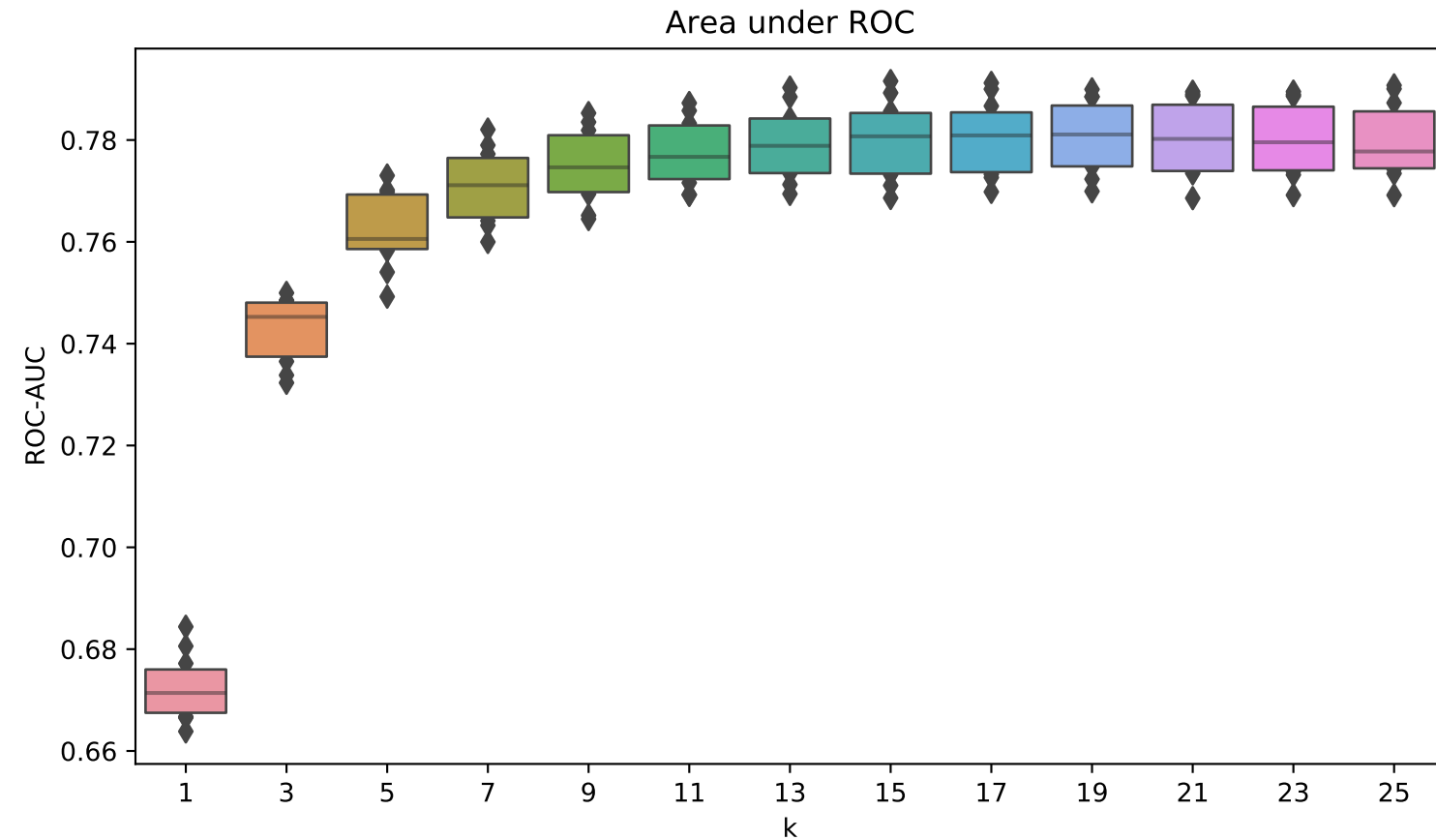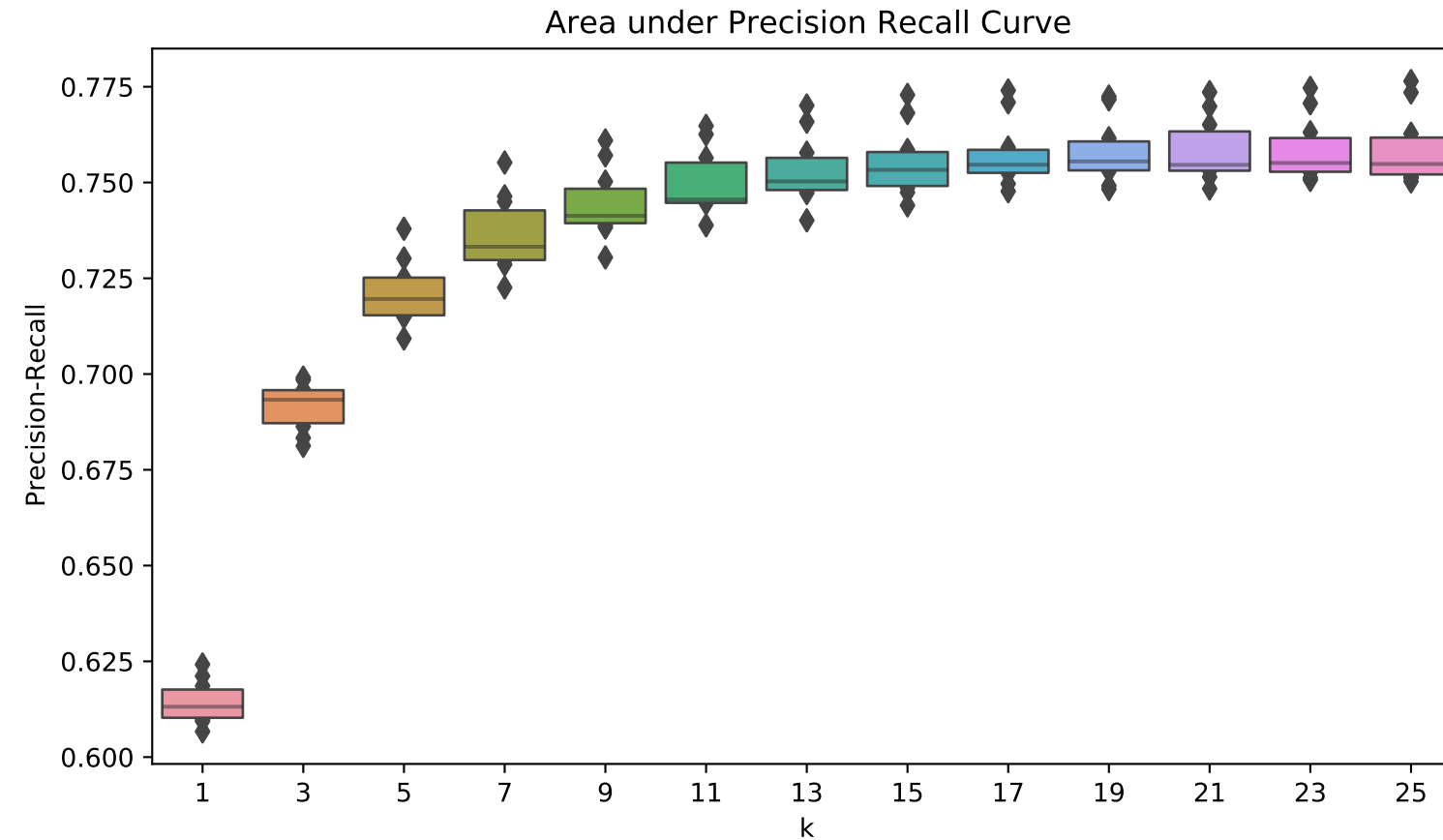

Supplement: btab294_Supplementary_Data [file btab294_supplementary_data.zip › btab294-suppl_data/weber.98.sup.fig.1.pdf]

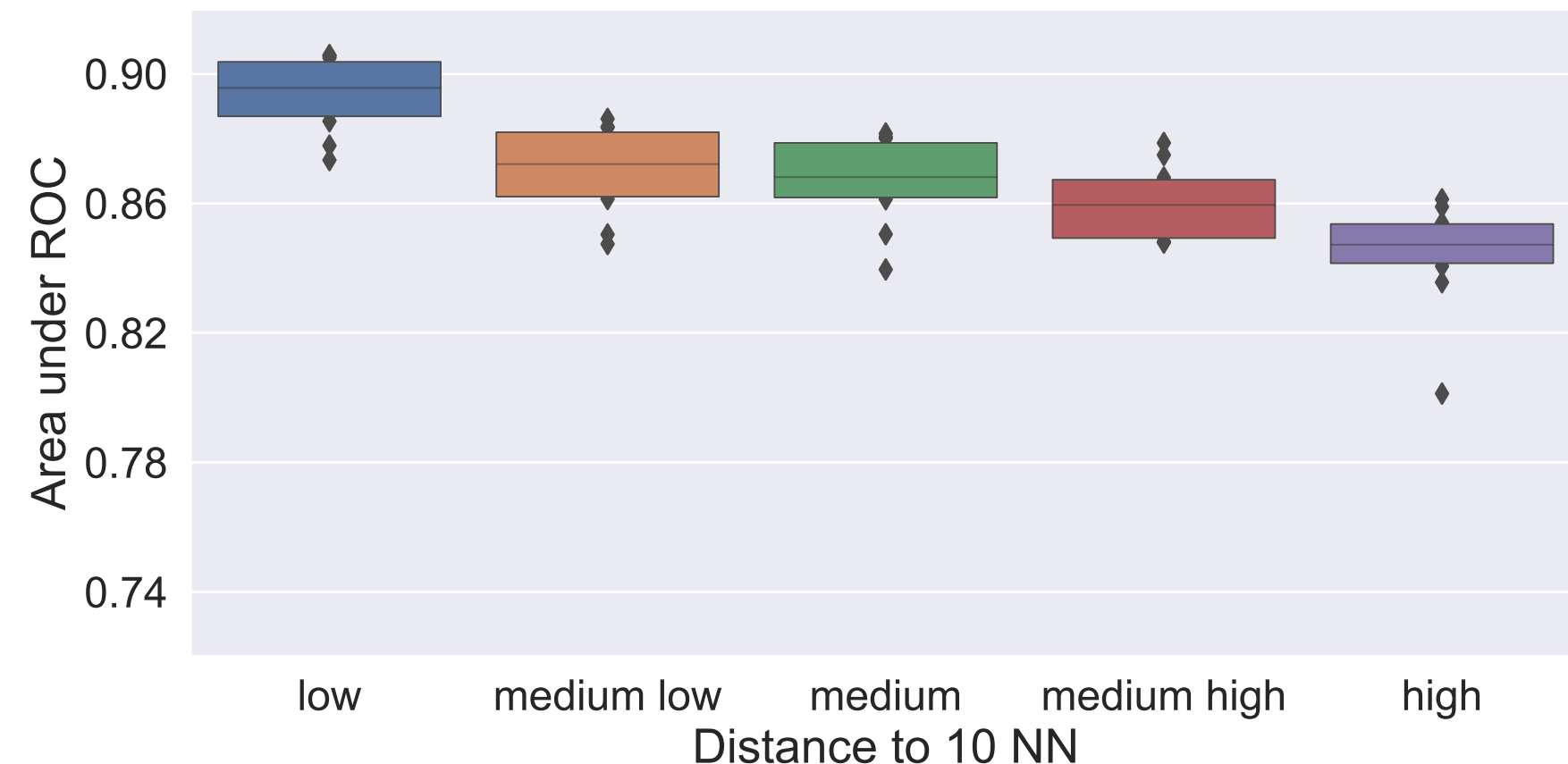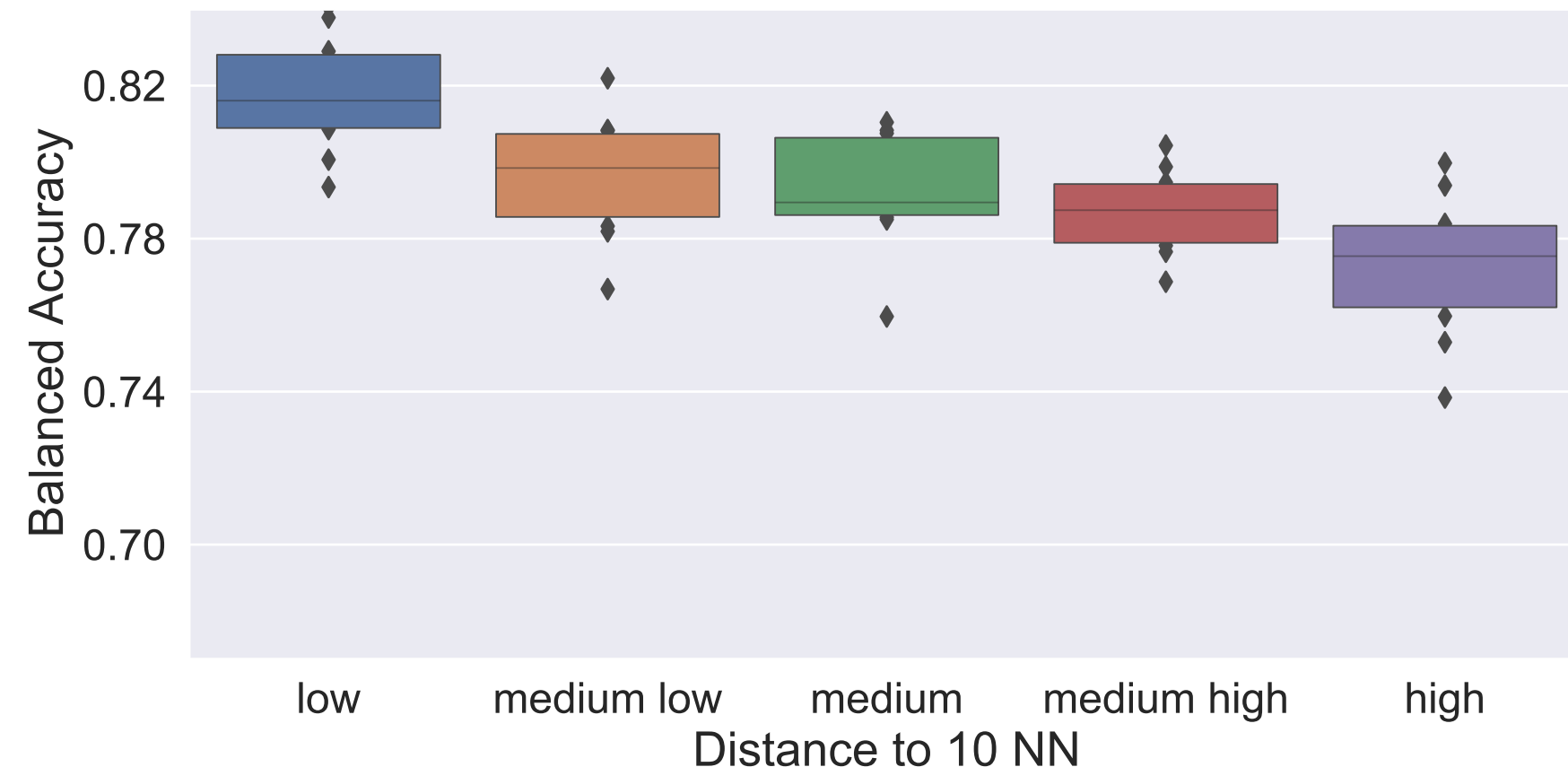

Supplement: btab294_Supplementary_Data [file btab294_supplementary_data.zip › btab294-suppl_data/weber.98.sup.fig.3.pdf]
